# Supplementary material for: Comparison of infectious complications with BCMA-directed therapies in multiple myeloma
Source: Blood Cancer J. 2024 May 31;14(1):88. doi: 10.1038/s41408-024-01043-5 (PMC11143331; doi:10.1038/s41408-024-01043-5)
Supplement: Supplementary file 1 — Supplemental Material [file 41408_2024_1043_MOESM1_ESM.docx]

**Supplemental Material**

**Comparison of Infectious Complications with BCMA-directed Bispecific Antibodies versus CAR T-Cells in Multiple Myeloma**

Karthik Nath, Tala Shekarkhand, David Nemirovsky, Andriy Derkach, Bruno Almeida Costa, Noriko Nishimura, Tasmin Farzana, Colin Rueda, David J. Chung, Heather J. Landau, Oscar B. Lahoud, Michael Scordo, Gunjan L. Shah, Hani Hassoun, Kylee Maclachlan, Neha Korde, Urvi A. Shah, Carlyn R. Tan, Malin Hultcrantz, Sergio A. Giralt, Saad Z. Usmani, Zainab Shahid, Sham Mailankody and Alexander M. Lesokhin

**Table of Contents**

Supplemental Table 1.…………………………………………………………………..………..Page 3

Supplemental Table 2………….………………………………………………………..………..Page 5

Supplemental Table 3………….………………………………………………………..………..Page 6

Supplemental Table 4………….………………………………………………………..………..Page 7

Supplemental Table 5………….………………………………………………………..………..Page 9

Supplemental Table 6….………………………………………………………………………..Page 10

Supplemental Table 7…………...……………………………………………………..………..Page 12

Supplemental Table 8………………...…………………………………………………………Page 13

Supplemental Table 9………………...…………………………………………………………Page 15

**Supplemental Table 1a**. Baseline characteristics of patients in the BsAb arm after excluding those who received prior CAR-T.

| Characteristic | Cohort | | p-value*^2^* | |
| --- | --- | --- | --- | --- |
|  | **BsAb, N = 37***^1^* | **CAR-T, N = 92***^1^* | |  |
| Age |  |  | | **0.042** |
| Median (IQR) | 66 (59, 75) | 62 (56, 69) | |  |
| Isotype |  |  | | >0.9 |
| Kappa | 21 (57%) | 52 (57%) | |  |
| Lambda | 16 (43%) | 40 (43%) | |  |
| Triple/Penta Refractory |  |  | | 0.6 |
| Neither | 6 (16%) | 20 (22%) | |  |
| Triple | 31 (84%) | 72 (78%) | |  |
| Penta | 16 (43%) | 32 (35%) | |  |
| Number of Prior Lines |  |  | | 0.069 |
| Median (IQR) | 5 (4, 8) | 6.5 (5, 8.2) | |  |
| Prior BCMA | 8 (22%) | 12 (13%) | | 0.2 |
| Prior ADC | 8 (22%) | 11 (12%) | | 0.2 |
| Prior CAR | 0 (0%) | 0 (0%) | |  |
| Prior Auto | 24 (65%) | 89 (97%) | | **<0.001** |
| Prior Allo | 1 (2.7%) | 5 (5.4%) | | 0.7 |
| Lymphopenia (Gr 3+) | 10 (27%) | 21 (23%) | | 0.6 |
| Neutropenia (Gr 3+) | 2 (5.4%) | 1 (1.1%) | | 0.2 |
| Hypogammaglobulinemia | 18 (50%) | 55 (60%) | | 0.3 |
| Time on Follow-Up (mo) |  |  | |  |
| Median (IQR) | 4.7 (3.5, 10.8) | 5.8 (3.8, 9.2) | |  |
| *^1^* n (%) | | | | |
| *^2^* Wilcoxon rank sum test; Pearson's Chi-squared test; Fisher's exact test | | | | |

**Supplemental Table 1b**. Baseline characteristics of patients in the BsAb arm who received prior CAR-T.

| Characteristic | BsAb, N = 18 |
| --- | --- |
| Age |  |
| Median (IQR) | 65 (57, 70) |
| Isotype |  |
| Kappa | 10 (56%) |
| Lambda | 8 (44%) |
| Triple/Penta Refractory |  |
| Neither | 0 (0%) |
| Triple | 4 (22%) |
| Penta | 14 (78%) |
| Number of Prior Lines |  |
| Median (IQR) | 9 (6, 10) |
| Prior BCMA | 17 (94%) |
| Prior ADC | 11 (61%) |
| Prior CAR | 18 (100%) |
| Prior Auto | 17 (94%) |
| Prior Allo | 2 (11%) |
| Lymphopenia (Gr 3+) | 11 (61%) |
| Neutropenia (Gr 3+) | 1 (5.6%) |
| Hypogammaglobulinemia | 8 (50%) |
| Time on Follow-Up (mo) |  |
| Median (IQR) | 4.0 (2.6, 4.8) |

**Supplemental Table 2**. Multivariable analysis of baseline factors and risk of any-grade infection.

| Characteristic | N |  | Event | HR*^1^* | 95% CI*^1^* | p-value | |
| --- | --- | --- | --- | --- | --- | --- | --- |
| Cohort |  |  |  |  |  | |  |
| BsAb | 53 |  | 39 | Ref. | Ref. | |  |
| CAR-T | 92 |  | 55 | 0.73 | 0.48, 1.10 | | 0.13 |
| Age | 145 |  | 94 | 1.00 | 0.98, 1.02 | | >0.9 |
| Triple/Penta Refractory |  |  |  |  |  | |  |
| Neither | 25 |  | 15 | Ref. | Ref. | |  |
| Triple | 59 |  | 42 | 1.20 | 0.68, 2.13 | | 0.5 |
| Penta | 61 |  | 37 | 1.22 | 0.66, 2.26 | | 0.5 |
| Prior BCMA therapy | 145 |  | 94 | 0.65 | 0.40, 1.07 | | 0.091 |
| Neutropenia (G3+) | 145 |  | 94 | 2.29 | 1.49, 3.51 | | **<0.001** |
| Lymphopenia (G3+) | 145 |  | 94 | 0.90 | 0.55, 1.47 | | 0.7 |
| Hypogammaglobulinemia | 145 |  | 94 | 1.01 | 0.64, 1.60 | | >0.9 |

*^1^*HR = Hazard Ratio, CI = Confidence Interval

**Supplemental Table 3**. Baseline characteristics of patients treated with antibody drug conjugates.

| Characteristic | N = 109*^1^* |
| --- | --- |
| Age |  |
| Median (IQR) | 67 (61, 73) |
| Isotype |  |
| Kappa | 65 (60%) |
| Lambda | 43 (40%) |
| Triple/Penta Refractory |  |
| Neither | 17 (16%) |
| Triple | 50 (46%) |
| Penta | 42 (39%) |
| Number of Prior Lines |  |
| Median (IQR) | 6 (5, 8) |
| Prior BCMA | 19 (18%) |
| Prior ADC | 1 (0.9%) |
| Prior CAR-T | 17 (16%) |
| Prior Auto transplant | 85 (78%) |
| Prior Allo transplant | 5 (4.6%) |
| Lymphopenia (G3+) | 32 (30%) |
| Neutropenia (G3+) | 9 (8.3%) |
| Hypogammaglobulinemia | 63 (58%) |

n (%)

**Supplemental Table 4A.** Differences in severe (grade ≥3) infection rate between cohorts (with BsAb as the reference group) during periods of treatment-emergent hypogammaglobulinemia using Poisson regression.

| **Characteristic** | **Incidence rate ratio (IRR)** | **95% CI** | **P-value** |
| --- | --- | --- | --- |
| **100-day follow-up** | | | |
| CAR-T vs BsAb | 1.18 | 0.51 – 3.05 | 0.7 |
| **6-month follow-up** | | | |
| CAR-T vs BsAb | 0.61 | 0.33 – 1.14 | 0.11 |
| **1-year follow-up** | | | |
| **All infection types** | | | |
| CAR-T vs BsAb | 0.44 | 0.25 – 0.76 | **0.004** |
| **Bacterial infections** | | | |
| CAR-T vs BsAb | 0.42 | 0.21 – 0.83 | **0.013** |
| **Viral infections** | | | |
| CAR-T vs BsAb | 0.61 | 0.22 – 1.75 | 0.3 |

**Supplemental Table 4B.** Differences in severe (grade ≥3) infection rate between cohorts (with BsAb as the reference group) during periods of non-hypogammaglobulinemia Poisson regression.

| **Characteristic** | **IRR** | **95% CI** | **P-value** |
| --- | --- | --- | --- |
| **100-day follow-up** | | | |
| CAR-T vs BsAb | 0.93 | 0.22 – 3.95 | > 0.9 |
| **6-month follow-up** | | | |
| CAR-T vs BsAb | 0.66 | 0.18 – 2.37 | 0.5 |
| **1-year follow-up** | | | |
| **All infection types** | | | |
| CAR-T vs BsAb | 0.46 | 0.13 – 1.54 | 0.2 |
| **Bacterial infections** | | | |
| CAR-T vs BsAb | 0.74 | 0.16 – 3.77 | 0.7 |
| **Viral infections** | | | |
| CAR-T vs BsAb | 0.28 | 0.01 – 2.90 | 0.3 |

**Supplemental Table 4C.** Effects of hypogammaglobulinemia (HG), with non-hypogammaglobulinemia as the reference group, on severe (grade 3+) infection rate, within each cohort, using mixed effects Poisson regression with a patient-specific random effect.

|  | **BsAb Cohort** | | | **CAR-T Cohort** | | |
| --- | --- | --- | --- | --- | --- | --- |
| **Characteristic** | **IRR** | **95% CI** | **P-value** | **IRR** | **95% CI** | **P-value** |
| **100-day follow-up** |  | | |  | | |
| HG vs Non-HG | 0.80 | 0.24 – 2.75 | 0.7 | 1.02 | 0.34 – 3.02 | > 0.9 |
| **6-month follow-up** |  | | |  | | |
| HG vs Non-HG | 1.21 | 0.42 – 3.44 | 0.7 | 1.13 | 0.43 – 2.98 | 0.8 |
| **1-year follow-up** |  | | |  | | |
| **All infection types** |  | | |  | | |
| HG vs Non-HG | 1.51 | 0.58 – 3.90 | 0.4 | 1.51 | 0.50 – 4.56 | 0.5 |
| **Bacterial infections** |  | | |  | | |
| HG vs Non-HG | 2.00 | 0.53 – 7.46 | 0.3 | 1.14 | 0.29 – 4.42 | 0.8 |
| **Viral infections** |  | | |  | | |
| HG vs Non-HG | 1.27 | 0.18 – 8.89 | 0.8 | 2.49 | 0.31 – 19.9 | 0.4 |

**Supplemental Table 5A.** Effects of hypogammaglobulinemia (HG) on time to severe (grade 3+) infection, within each cohort, using cause-specific Cox regression.

|  | **BsAb Cohort** | | | | **CAR-T Cohort** | | | |
| --- | --- | --- | --- | --- | --- | --- | --- | --- |
| **Characteristic** | **Event (N)** | **HR** | **95% CI** | **P-value** | **Event (N)** | **HR** | **95% CI** | **P-value** |
| Non-HG | 5 | Ref. | Ref. |  | 6 | Ref. | Ref. |  |
| HG | 17 | 1.37 | 0.46 – 4.07 | 0.6 | 18 | 1.44 | 0.53 – 3.93 | 0.5 |

**Supplemental Table 5B.** Differences in time to severe (grade 3+) infection between cohorts, during periods of treatment-emergent hypogammaglobulinemia and non-hypogammaglobulinemia, using cause-specific Cox regression.

|  | **Hypogammaglobulinemia Period** | | | | **Non-hypogammaglobulinemia Period** | | | |
| --- | --- | --- | --- | --- | --- | --- | --- | --- |
| **Characteristic** | **Event (N)** | **HR** | **95% CI** | **P-value** | **Event (N)** | **HR** | **95% CI** | **P-value** |
| BsAb | 17 | Ref. | Ref. |  | 5 | Ref. | Ref. |  |
| CAR-T | 18 | 0.49 | 0.25 – 0.96 | **0.036** | 6 | 0.77 | 0.21 – 2.78 | 0.7 |

**Supplemental Table 5C.** Differences in time to (any grade) infection between cohorts, during periods of treatment-emergent hypogammaglobulinemia using cause-specific Cox regression.

| **Characteristic** | **Event (N)** | **HR** | **95% CI** | **P-value** |
| --- | --- | --- | --- | --- |
| BsAb | 32 | Ref. | Ref. |  |
| CAR-T | 34 | 0.51 | 0.31 – 0.83 | **0.007** |

**Supplemental Table 6A.** Effects of neutropenia, with non-neutropenia as the reference group, on severe (grade 3+) infection rate, within each cohort, using mixed effects Poisson regression with a patient-specific random effect.

|  | **BsAb Cohort** | | | **CAR-T Cohort** | | |
| --- | --- | --- | --- | --- | --- | --- |
| **Characteristic** | **IRR** | **95% CI** | **P-value** | **IRR** | **95% CI** | **P-value** |
| **100-day follow-up** |  | | |  | | |
| Neutropenia vs Non-neutropenia | 3.45 | 0.75 – 16.0 | 0.11 | 2.68 | 1.14 – 6.31 | **0.024** |
| **6-month follow-up** |  | | |  | | |
| Neutropenia vs Non-neutropenia | 2.93 | 0.93 – 9.23 | 0.066 | 3.40 | 1.47 – 7.83 | **0.004** |
| **1-year follow-up** |  | | |  | | |
| **All infection types** |  | | |  | | |
| Neutropenia vs Non-neutropenia | 2.85 | 1.02 – 7.96 | **0.046** | 4.32 | 1.85 – 10.1 | **<0.001** |
| **Bacterial infections** |  | | |  | | |
| Neutropenia vs Non-neutropenia | 2.48 | 0.66 – 9.37 | 0.2 | 4.56 | 1.52 – 13.7 | **0.007** |
| **Viral infections** |  | | |  | | |
| Neutropenia vs Non-neutropenia | 2.22 | 0.18 – 28.2 | 0.5 | 3.74 | 1.00 – 13.9 | **0.049** |

**Supplemental Table 6B.** Differences in severe (grade 3+) infection rate between cohorts (with BsAb as the reference group) during periods of treatment-emergent neutropenia using Poisson regression.

| **Characteristic** | **IRR** | **95% CI** | **P-value** |
| --- | --- | --- | --- |
| **100-day follow-up** | | | |
| CAR-T vs BsAb | 0.67 | 0.18 – 4.30 | 0.6 |
| **6-month follow-up** | | | |
| CAR-T vs BsAb | 0.54 | 0.19 – 1.90 | 0.3 |
| **1-year follow-up** | | | |
| **All infection types** | | | |
| CAR-T vs BsAb | 0.49 | 0.18 – 1.53 | 0.2 |
| **Bacterial infections** | | | |
| CAR-T vs BsAb | 0.57 | 0.17 – 2.55 | 0.4 |
| **Viral infections** | | | |
| CAR-T vs BsAb | 0.75 | 0.11 – 14.7 | 0.8 |

**Supplemental Table 6C.** Differences in severe (grade 3+) infection rate between cohorts (with BsAb as the reference group) during periods of non-neutropenia using Poisson regression.

| **Characteristic** | **IRR** | **95% CI** | **P-value** |
| --- | --- | --- | --- |
| **100-day follow-up** | | | |
| CAR-T vs BsAb | 0.86 | 0.35 – 2.16 | 0.7 |
| **6-month follow-up** | | | |
| CAR-T vs BsAb | 0.47 | 0.23 – 0.94 | **0.033** |
| **1-year follow-up** | | | |
| **All infection types** |  |  |  |
| CAR-T vs BsAb | 0.32 | 0.17 – 0.59 | **<0.001** |
| **Bacterial infections** | | | |
| CAR-T vs BsAb | 0.33 | 0.15 – 0.69 | **0.004** |
| **Viral infections** | | | |
| CAR-T vs BsAb | 0.39 | 0.12 – 1.16 | 0.10 |

**Supplemental Table 7A.** Differences in time to severe (grade 3+) infection between cohorts, during periods of treatment-emergent neutropenia and non- neutropenia, using cause-specific Cox regression.

|  | **Neutropenia Period** | | | | **Non-neutropenia Period** | | | |
| --- | --- | --- | --- | --- | --- | --- | --- | --- |
| **Characteristic** | **Event (N)** | **HR** | **95% CI** | **P-value** | **Event (N)** | **HR** | **95% CI** | **P-value** |
| BsAb | 3 | Ref. | Ref. |  | 19 | Ref. | Ref. |  |
| CAR-T | 11 | 0.75 | 0.18 – 3.09 | 0.7 | 13 | 0.44 | 0.21 – 0.93 | **0.032** |

**Supplemental Table 7B.** Effects of neutropenia on time to severe (grade 3+) infection, within each cohort, using cause-specific Cox regression.

|  | **BsAb Cohort** | | | | **CAR-T Cohort** | | | |
| --- | --- | --- | --- | --- | --- | --- | --- | --- |
| **Characteristic** | **Event (N)** | **HR** | **95% CI** | **P-value** | **Event (N)** | **HR** | **95% CI** | **P-value** |
| Non-neutropenia | 19 | Ref. | Ref. |  | 13 | Ref. | Ref. |  |
| Neutropenia | 3 | 1.63 | 0.46 – 5.82 | 0.5 | 11 | 1.69 | 0.68 – 4.21 | 0.3 |

**Supplemental Table 8A**. Affected organ systems, grades, and infection types in CAR-T recipients.

| **Organ** | **CAR-T, N = 115***^1^* |
| --- | --- |
| Blood | 17 (15%) |
| Upper Respiratory | 46 (40%) |
| Lower Respiratory | 16 (14%) |
| Gastrointestinal | 18 (16%) |
| Genitourinary | 13 (11%) |
| Skin/Soft Tissue | 8 (7.0%) |
| Other (e.g., Bone, CNS, Cardiac) | 3 (2.6%) |
| **Grade of Infection** |  |
| 5 | 0 (0%) |
| 4 | 0 (0%) |
| 3 | 32 (28%) |
| ≤2 | 83 (72%) |
| **Infection Type** |  |
| Bacterial | 53 (46%) |
| Bacterial and Viral | 3 (2.6%) |
| Fungal | 5 (4.4%) |
| Parasitic | 1 (0.9%) |
| Viral | 52 (46%) |
| Unknown | 1 |
| *^1^*n (%) |  |

**Supplemental Table 8B**. Affected organ systems, grades, and infection types in BsAb recipients.

| **Organ** | **BsAb, N = 99***^1^* |
| --- | --- |
| Blood | 9 (9.1%) |
| Upper Respiratory | 49 (49%) |
| Lower Respiratory | 13 (13%) |
| Gastrointestinal | 9 (9.1%) |
| Genitourinary | 9 (9.1%) |
| Skin/Soft Tissue | 4 (4.0%) |
| Other (e.g., Bone, CNS, Cardiac) | 5 (5.1%) |
| **Grade of Infection** |  |
| 5 | 4 (4.0%) |
| 4 | 2 (2.0%) |
| 3 | 30 (30%) |
| ≤2 | 63 (64.1%) |
| **Infection Type** |  |
| Bacterial | 52 (54%) |
| Bacterial and Viral | 0 (0%) |
| Fungal | 3 (3.1%) |
| Parasitic | 0 (0%) |
| Viral | 42 (43%) |
| Unknown | 2 |
| *^1^*n (%)  **Supplemental Table 9A**. Infectious organisms identified in CAR-T recipients. |  |
| **Infectious Organism** | **CAR-T, N = 115^1^** |
| Astrovirus | 1 (0.9%) |
| Bacillus cereus | 1 (0.9%) |
| BK virus | 1 (0.9%) |
| Candida albicans | 6 (5.2%) |
| Chryseobacterium indologenes | 1 (0.9%) |
| Citrobacter freundii | 1 (0.9%) |
| Clostridioides difficile | 12 (10.4%) |
| Clostridium | 1 (0.9%) |
| Cytomegalovirus | 1 (0.9%) |
| Coagulase-negative staphylococci | 2 (1.7%) |
| COVID-19 | 4 (3.5%) |
| Cyclospora | 1 (0.9%) |
| E. Coli esbl | 1 (0.9%) |
| EBV | 1 (0.9%) |
| Enterococcus faecalis | 1 (0.9%) |
| Escherichia coli | 6 (5.2%) |
| Haemophilus influenzae | 1 (0.9%) |
| HHV-6 | 1 (0.9%) |
| HSV-1 | 1 (0.9%) |
| Human metapneumovirus | 2 (1.7%) |
| Influenza A | 2 (1.7%) |
| Influenza | 1 (0.9%) |
| Klebsiella pneumoniae | 3 (2.6%) |
| Legionella pneumophila | 1 (0.9%) |
| Metapneumovirus/rhinovirus | 1 (0.9%) |
| Micrococcus luteus | 1 (0.9%) |
| Moraxella catarrhalis | 1 (0.9%) |
| Morganella morganii | 1 (0.9%) |
| Pantoea agglomerans | 1 (0.9%) |
| Parainfluenza type 1 | 1 (0.9%) |
| Parainfluenza type 3 | 4 (3.5%) |
| Polymicrobial | 1 (0.9%) |
| Pseudomonas aeruginosa | 1 (0.9%) |
| Rhinovirus | 1 (0.9%) |
| Rhinovirus/enterovirus | 16 (14%) |
| Rhinovirus/enterovirus, possible superimposed bacterial etiology | 1 (0.9%) |
| Rhinovirus/enterovirus; metapneumovirus | 1 (0.9%) |
| RSV | 4 (3.5%) |
| RSV; coronavirus type nl63 | 1 (0.9%) |
| RVP parainfluenza type 3 | 1 (0.9%) |
| Sapovirus | 1 (0.9%) |
| Sars‑cov‑2 | 1 (0.9%) |
| Sars‑cov‑2; adenovirus | 1 (0.9%) |
| Staphylococcus aureus | 1 (0.9%) |
| Staphylococcus epidermidis | 1 (0.9%) |
| Unknown | 20 (17%) |
| ^1^n (%) | |

**Supplemental Table 9B**. Infectious organisms identified in BsAb recipients.

| **Infectious Organism** | **BsAb, N = 99^1^** |
| --- | --- |
| Adenovirus | 2 (2.0%) |
| Campylobacter + norovirus | 1 (1.0%) |
| Candida albicans | 1 (1.0%) |
| Citrobacter freundii | 1 (1.0%) |
| Clostridioides difficile | 3 (3.0%) |
| Clostridium innocuum | 1 (1.0%) |
| Cornybacterium | 1 (1.0%) |
| Coronavirus type nl63 | 1 (1.0%) |
| COVID-19 | 8 (8.1%) |
| Cytomegalovirus | 2 (2.0%) |
| Varicella-zoster | 2 (2.0%) |
| E.coli | 5 (5.1%) |
| Enterobacter cloacae/asburiae | 1 (1.0%) |
| Enterococcus faecalis + proteus mirabilis | 1 (1.0%) |
| Hepatitis B reactivation | 1 (1.0%) |
| HSV-2 | 1 (1.0%) |
| Influenza A | 4 (4.0%) |
| Klebsiella aerogenes | 1 (1.0%) |
| Klebsiella oxytoca | 1 (1.0%) |
| Klebsiella pneumoniae | 4 (4.0%) |
| Metapneumovirus | 2 (2.0%) |
| Norovirus | 2 (2.0%) |
| Oral candidiasis | 1 (1.0%) |
| Parvovirus | 1 (1.0%) |
| Pneumocystis | 1 (1.0%) |
| Pseudomonas | 2 (2.0%) |
| Pseudomonas aeruginosa | 3 (3.0%) |
| Rhinovirus | 14 (14%) |
| Serratia marcescens | 1 (1.0%) |
| Stenotrophomonas maltophilia | 1 (1.0%) |
| Streptococcus mitis group | 1 (1.0%) |
| Unknown | 28 (28%) |
| ^1^n (%) |  |
